# Vascular Biology of Superoxide-Generating NADPH Oxidase 5—Implications in Hypertension and Cardiovascular Disease

**DOI:** 10.1089/ars.2018.7583

**Published:** 2019-01-21

**Authors:** Rhian M. Touyz, Aikaterini Anagnostopoulou, Livia L. Camargo, Francisco J. Rios, Augusto C. Montezano

**Affiliations:** BHF Glasgow Cardiovascular Research Centre, Institute of Cardiovascular and Medical Sciences, University of Glasgow, Glasgow, United Kingdom.

**Keywords:** Nox isoforms, vascular smooth muscle, endothelium, reactive oxygen species, oxidative stress, atherosclerosis

## Abstract

***Significance:*** NADPH oxidases (Noxs), of which there are seven isoforms (Nox1–5, Duox1/Duox2), are professional oxidases functioning as reactive oxygen species (ROS)-generating enzymes. ROS are signaling molecules important in physiological processes. Increased ROS production and altered redox signaling in the vascular system have been implicated in the pathophysiology of cardiovascular diseases, including hypertension, and have been attributed, in part, to increased Nox activity.

***Recent Advances:*** Nox1, Nox2, Nox4, and Nox5 are expressed and functionally active in human vascular cells. While Nox1, Nox2, and Nox4 have been well characterized in models of cardiovascular disease, little is known about Nox5. This may relate to the lack of experimental models because rodents lack *NOX5*. However, recent studies have advanced the field by (i) elucidating mechanisms of Nox5 regulation, (ii) identifying Nox5 variants, (iii) characterizing Nox5 expression, and (iv) discovering the Nox5 crystal structure. Moreover, studies in human Nox5-expressing mice have highlighted a putative role for Nox5 in cardiovascular disease.

***Critical Issues:*** Although growing evidence indicates a role for Nox-derived ROS in cardiovascular (patho)physiology, the exact function of each isoform remains unclear. This is especially true for Nox5.

***Future Directions:*** Future directions should focus on clinically relevant studies to discover the functional significance of Noxs, and Nox5 in particular, in human health and disease. Two important recent studies will impact future directions. First, Nox5 is the first Nox to be crystallized. Second, a genome-wide association study identified Nox5 as a novel blood pressure-associated gene. These discoveries, together with advancements in Nox5 biology and biochemistry, will facilitate discovery of drugs that selectively target Noxs to interfere in uncontrolled ROS generation.

## An Introduction to Reactive Oxygen Species and Cell Biology

Reactive oxygen species (ROS) are signaling molecules that influence gene expression, oxygen sensing, cell growth, and cell death. They define the cellular phenotype by regulating functions such as migration, contraction, differentiation, and secretion (109). ROS have been implicated in many biological processes, including immune cell activation, bone reabsorption, sperm–oocyte fusion, aging, and regulation of vascular tone (87, 129).

The most well-known function of ROS relates to host defense responses, where superoxide production by phagocytes plays a key role in killing of microorganisms (118). The phagocyte respiratory burst oxidase, NADPH oxidase (Nox), catalyzes NADPH-dependent reduction of molecular O_2_ to produce superoxide (NADPH +2O_2_ → NADP^+^ + H^+^ + 2O_2_^−^), which can dismute to generate secondary species, including hydrogen peroxide (H_2_O_2_) and hypochlorous acid, which together play a role in host defense by killing or damaging microbes (9, 111). Phagocytic Nox comprises two plasma membrane-associated subunits (gp91phox [also called Nox2] and p22phox), three cytosolic subunits (p47phox, p67phox, and p40phox), and a low-molecular-weight G protein (Rac1 or Rac2), which are disassociated in the resting state (77, 97). Upon stimulation, assembly of the plasma membrane subunits with cytosolic subunits and Rac1/2 leads to superoxide generation. The catalytic subunit is Nox2, which forms a transmembrane heterodimer with p22phox and functions as an electron transport chain. Phagocytic Nox has been very well described and its critical role in clinical medicine is highlighted in patients with chronic granulomatous disease (CGD) (14, 22), which is caused by a deficiency in one of the phox subunits. Patients with CGD are unable to generate superoxide and the disease is characterized by severe, and often fatal, infections that require bone marrow transplant to reduce clinical symptoms (43).

Nox, in particular Nox2, the Nox prototype (23, 25), was discovered in the 1960s, with studies demonstrating its functional activity in phagocytic cells such as neutrophils and monocytes/macrophages. Interestingly, some of the earliest studies actually identified Nox in nonphagocytic cells, including the liver, heart, and brain (9, 91, 98). However, it has only been in the past 20 years that the importance of nonphagocytic Nox-derived ROS in physiological and pathological processes has become evident. It is now clear that various cell types, including kidney cells, fibroblasts, adipocytes, spermatozoa, osteoclasts, thyroid, brain, colon, cardiomyocytes, and hepatocytes, as well as vascular cells, generate ROS (60, 69, 79, 89, 116, 122). Although many enzymatic sources, such as mitochondria, peroxisomes, and cytochrome P450, can produce ROS in these cells, Nox is especially important as its primary function is ROS generation, whereas other enzymes produce ROS as by-products of enzymatic activity (21).

Since the discovery of Nox2, six other Nox isoforms have been identified, including Nox1, Nox3, Nox4, Nox5, Duox1, and Duox2 (83, 115). The existence of multiple homologs of Nox2 in nonphagocytic cells/tissues suggests that production of ROS in these sites is distinct and different from host defense functions (6).

Although there has been enormous advancement in the field of Nox biology, the specific biological significance and mechanisms of regulation of the different Noxs still remain elusive (6). Rodent vascular, cardiac, and renal cells express Nox1, Nox2, and Nox4, and in humans, Nox5 is also present (64, 85, 107). These Noxs have been implicated in many redox-sensitive pathophysiological processes in the heart and vascular and renal systems (64, 85, 107) and have been suggested to be a cause of altered redox status in cardiovascular disease, including hypertension and atherosclerosis.

## ROS and Hypertension

Mechanisms contributing to hypertension are complex, involving many pathological processes, including altered renal Na^+^ regulation, endothelial dysfunction, vascular remodeling, vascular hypercontractility, arterial calcification, immune cell activation, and inflammation (106, 114). Common to these processes is oxidative stress due to excessive Nox-derived ROS generation, reduced nitric oxide (NO) bioavailability, and decreased antioxidant capacity (31). Oxidative stress is associated with increased redox signaling, activation of transcription factors, stimulation of proinflammatory and profibrotic signaling pathways, induction of stress-related kinases and DNA damage, molecular processes that lead to cell damage, and vascular dysfunction (49).

Abnormal redox signaling and oxidative stress have been shown to play a role in development of hypertension in experimental models. In angiotensin II (Ang II)-induced hypertension in rodents, production of O_2_^−^ and H_2_O_2_ is increased in vascular, renal, cardiac, and neural cells and antioxidant enzymatic activity is reduced (13, 26, 71, 95, 96). Oxidative stress has also been demonstrated in salt-sensitive forms of hypertension (29). Rats with deoxycorticosterone acetate salt-induced mineralocorticoid hypertension exhibit elevated vascular O_2_^−^ production due to activation of Noxs and mitochondrial oxidases and uncoupling of endothelial nitric oxide synthase (110, 133). Nox1-dependent ROS production has also been demonstrated in experimental models of pulmonary hypertension (56) and seems to be especially important in arterial fibrosis and vascular aging in stroke-prone spontaneously hypertensive rats (52).

While extensive experimental data support a causative role for oxidative stress in hypertension, there is a paucity of evidence for a direct role of ROS in the pathophysiology of human hypertension (82, 84, 101). However, many clinical studies have demonstrated a positive association between blood pressure and plasma levels of biomarkers of oxidative stress, including malondialdehyde, F2-isoprostanes, and advanced oxidation protein products, and an inverse association between blood pressure and plasma antioxidants, including total antioxidant capacity, superoxide dismutase (SOD), and antioxidant vitamins (vitamins E and C) (35, 101, 120). Despite these strong suggestions linking oxidative stress and human hypertension, further studies are still required to unambiguously demonstrate a causal relationship. Moreover, to date, there are still no convincing data supporting a role for antioxidants in the prevention or treatment of essential hypertension (47, 104).

Of the many ROS-generating enzymes in the cardiovascular system, Noxs appear to be especially important in the pathophysiology of hypertension. A direct causal role for Noxs in the development of hypertension has been demonstrated in mice lacking Nox1, Nox2, Nox4, or p47phox where Ang II-induced endothelial dysfunction and hypertension are blunted (100, 124). While acute models of Ang II-mediated hypertension involve Nox activation, chronic models of Ang II-dependent hypertension seem to be independent of Nox1 and Nox2 (119, 131). Transgenic mice overexpressing Noxs in the vascular wall have exaggerated vascular and blood pressure responses to Ang II (100). In Ang II-infused mice overexpressing p22phox in a vascular smooth muscle cell-specific manner, vascular hypertrophy and pressor effects were exacerbated compared with control mice (125a). These mice also exhibited an increase in NO and H_2_O_2_ generation (65). Treatment of hypertensive mice and rats with various antioxidants, including vitamins C and E, SOD, and tempol, reduced vascular and systemic oxidative stress and decreased blood pressure (11, 127). Strategies to increase NO, including treatment with BH4, have been shown to have antihypertensive and vasoprotective effects in models of experimental and pulmonary hypertension (15, 105). Treatment of hypertensive mice with Nox inhibitors, such as apocynin, diphenyleneiodonium, gp91ds-tat, GKT137831, or antioxidants, to reduce ROS bioavailability improved vascular function and normalized blood pressure, further supporting an important link between Nox activation, oxidative stress, and blood pressure elevation (19, 123). With increasing interest in development of Nox isoform-specific inhibitors, the need to fully understand the differential role of Noxs in vascular oxidative stress is growing. We provide an overview on current knowledge regarding the role of p22phox-dependent Noxs (Nox1, Nox2, and Nox4) in the vascular system and discuss in detail the importance of p22phox-dependent Noxs, specifically Nox5, which is emerging as an important Nox isoform in human disease.

## p22phox-Dependent Nox Isoforms in the Vascular System

p22phox, a nonglycosylated membrane-associated protein of molecular mass 22 kDa, associates with Nox in a 1:1 ratio (114). p22phox expression increases in response to Ang II and is upregulated in experimental diabetes and hypertension. Its major functions are to stabilize Nox proteins (Nox1, Nox2, Nox3, and Nox4) and to interact with cytosolic organizer subunits for Nox1, Nox2, and Nox3 (106). While Noxs 1–4 have an obligatory need for p22phox for activation, Nox5 does not require p22phox for activity (5).

Details about vascular Nox1, Nox2, and Nox4 have been extensively reviewed (8, 40, 68, 70, 90) and only an overview is given here for completeness. The message and protein for Nox1, Nox2, and Nox4 have been identified in arteries in rodents and humans. All Noxs are expressed to varying degrees in endothelial cells, vascular smooth muscle cells, perivascular adipocytes, and fibroblasts (36, 103, 112). Nox4 is abundant in endothelial cells. Nox1 and Nox2 are expressed in vascular smooth muscle cells, with Nox1 being predominant in large conduit arteries and Nox2 in peripheral arteries (68). Constitutively active Nox4 seems to play a role in basal generation of ROS.

The function of Nox-derived ROS in the vascular system is complex and depends on the target cell type, predominant Nox isoform, type of species generated, stimulating factor, and redox state. Ang II and other prohypertensive factors cause upregulation of vascular Nox1 and Nox2, important in redox-mediated hypertension in various experimental models. Vascular Nox1/2-derived ROS has also been suggested in atherosclerosis, diabetic vasculopathy, vascular remodeling, and aortic aneurysm (46, 113, 125, 126). The role of Nox4 is less clear because it has been shown to be both vasoinjurious and vasoprotective (8, 78), depending on experimental conditions. Endothelial Nox4 influences angiogenesis and vascular aging (67), and perivascular Nox4 is important in vascular remodeling in pulmonary hypertension (4). In ischemic or hypoxic conditions, endothelial Nox4 plays a role in blood–brain barrier breakdown and neural autotoxicity. Nox4^−/−^ mice were protected from oxidative stress, blood–brain barrier leakage, and neuronal apoptosis in a stroke model (12), indicating a pathological role of Nox4 in this model. Nox4 is also important in fibrosis and cardiovascular remodeling (63).

On the other hand, emerging evidence indicates vasoprotective and antiatherosclerotic functions for endothelial and cardiomyocyte Nox4 because deletion of Nox4 in these cell types is associated with worse cardiovascular injury and endothelial dysfunction in experimental models of atherosclerosis, cardiac ischemia, and hypertension (86, 134). Reasons for the discrepant results likely relate to the site of Nox4 activation, type and site of ROS production, and experimental models studied.

The exact role of Nox4 in humans is still unclear, although clinical studies imply that Nox4 is important in fibrosis. Various drugs and small-molecule inhibitors targeting Nox4 in pathological fibrosis are under development (117). A Nox1/4 inhibitor, GKT136901, is currently in Phase 2 clinical trials for treating hepatic fibrosis (71). Whether such approaches will be beneficial in cardiovascular fibrosis and remodeling is unclear.

## p22phox-Independent Nox Isoforms: Focus on Nox5

In addition to Noxs 1–4, three other Nox isoforms have been identified, including Nox5, Duox1, and Duox2, which share ≈50% homology with Nox2 (2, 27). These Noxs are unique, in that they possess a long NH_2_ terminus containing a Ca^2+^-binding EF-hand domain and do not require p22phox or cytosolic Nox subunits for their activation. Nox5 has a molecular mass of ≈85 kDa and does not seem to be glycosylated, whereas Duox1 and Duox2 have *N*-glycosylation sites and a molecular mass of 160–190 kDa (2). These Noxs are directly activated by Ca^2+^, which binds to the EF-hand Ca^2+^-binding domains, causing conformational change and activation of Nox to generate O_2_ (2, 3, 27).

### Duox1/2

Duox1 and Duox2 were originally identified in the thyroid gland and are critically involved in redox-dependent synthesis of thyroid hormone (10). Duox1/2 are highly expressed in the thyroid gland, but Duox1 has also been identified in low levels in respiratory epithelium, while Duox2 has been identified in gastrointestinal glandular epithelium, salivary glands, and tumors (32, 128). Little is known about Duoxs in the cardiovascular system. A single study reported that aortic smooth muscle cells express low levels of Duox1 mRNA (59); however, the function is unclear. In the heart, Duox-mediated H_2_O_2_ generation has been observed in zebrafish (51), but to our knowledge, nothing is known about Duox in the mammalian heart.

### Nox5

**FIG. 1.**
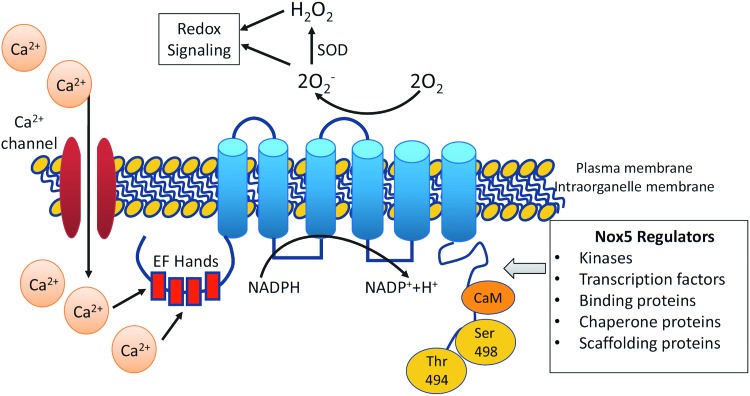
**Diagram demonstrating the structure and regulation of Nox5.** Nox5 possesses a six-transmembrane domain, N-terminal domain with EF-hands, and C-terminal domain with phosphorylation sites. Nox5 is regulated by changes in intracellular Ca^2+^ levels, regulated, in part, by Ca^2+^ influx through Ca^2+^ channels. Nox5 is regulated by various kinases, transcription factors, and binding proteins, which may influence phosphorylation of Nox5. Nox5 activation generates O_2_^−^, which is dismutated by SOD to H_2_O_2_. O_2_^−^ and H_2_O_2_ act as signaling molecules influencing redox-sensitive signaling pathways. H_2_O_2_, hydrogen peroxide; Nox, NADPH oxidase; O_2_^−^, superoxide; SOD, superoxide dismutase. To see this illustration in color, the reader is referred to the web version of this article at www.liebertpub.com/ars

Nox5, of which there are multiple isoforms (Nox5α, -β, -γ, and -ɛ [also called Nox5S]), is the most recently discovered Nox and is unique (Fig. 1) (20). Nox5ɛ is a short form of Nox5 and lacks Ca^2+^-binding domains. Unlike other isoforms, the Nox5 gene is absent in rodents, it generates ROS from a single gene product, and does not require Nox subunits for its activation. Its long, intracellular N-terminal domain with EF-hands undergoes conformational changes upon Ca^2+^ binding. Increased cytoplasmic Ca^2+^ concentration ([Ca^2+^]_i_) is essential for Nox5 activation, as evidenced in cell studies where ROS are not generated in Ca^2+^-free buffer (20, 71). Because of its close association with Ca^2+^, Nox5 is likely to be involved in Ca^2+^-activated redox-dependent processes. Nox5 regulation also depends on its gene expression, subcellular localization, and post-translational modifications. Of all the Nox isoforms, only Nox5 has recently been crystallized (72).

## Nox5 Compartmentalization and Regulation

**FIG. 2.**
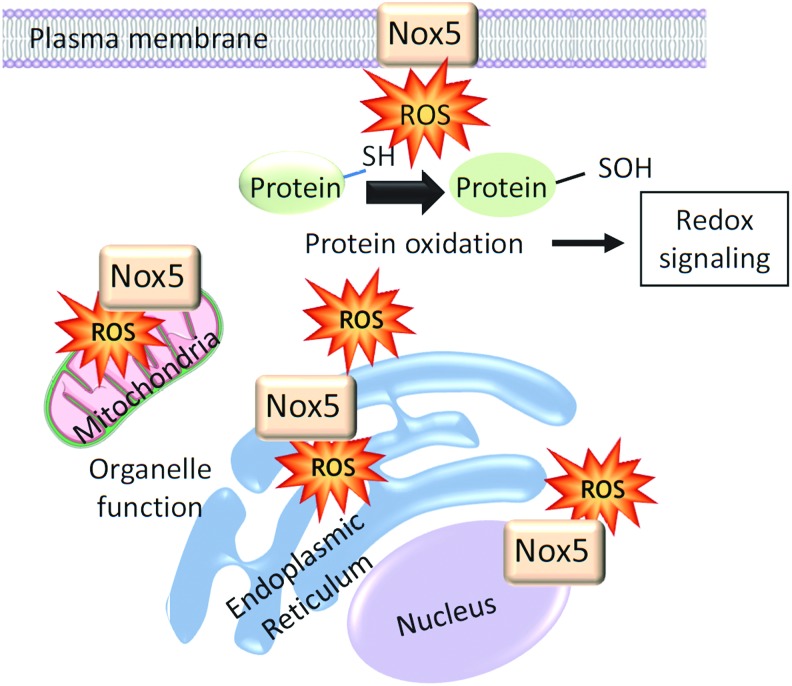
**Subcellular localization of Nox5.** Nox5 has been identified in the plasma membrane as well as various intracellular locations. It is highly expressed in the perinuclear area and is also detected in mitochondria and ER. The site-specific location of Nox5 may influence the function of this isoform through close association with specific signaling molecules. Increased Nox5-induced generation of ROS leads to oxidation of signaling molecules that influence redox signaling. ER, endoplasmic reticulum; ROS, reactive oxygen species. To see this illustration in color, the reader is referred to the web version of this article at www.liebertpub.com/ars

Unlike p22phox-regulated vascular Noxs (Nox1, Nox2, and Nox4), Nox5 is expressed primarily in intracellular compartments localized mainly in the perinuclear area and endoplasmic reticulum (ER) (Fig. 2) (66). In COS7-Nox5-transfected cells, Nox5 is also found in the Golgi and mitochondria, and in Nox5-overexpressing endothelial cells, Nox5 localizes in caveolae (66, 121). The significance of Nox5 in ER is unclear, but this is a site of Nox synthesis and post-translational modification. In addition, ER function is redox dependent and ER stress influences Nox5-ROS regulation (92). Hence, there may be bidirectional dependency. Nox5 seems to traffic from intracellular membranes to the plasma membrane where it may associate with cholesterol-rich microdomains, including caveolae and lipid rafts. The distinct intracellular distribution of Nox5 not only regulates ROS generation in a site-specific manner but also influences the function of redox-sensitive signaling molecules in close proximity, thereby controlling cell function.

In addition to its distinct subcellular localization, Nox5 activity is regulated, in part, by phosphorylation of serine/threonine (Ser475, Ser490, Ser494, and Ser498). Nox5 phosphorylation increases sensitivity to Ca^2+^, thereby facilitating ROS generation at lower Ca^2+^ levels (33). Numerous kinases, including protein kinase C (PKC)α, c-Abl, Ca^2+^/calmodulin-dependent protein kinase II (CAM kinase II), extracellular signal-regulated kinase 1/2 (ERK 1/2), and tyrosine, influence phosphorylation of Nox5 (Table 1) (16, 33, 108). Nox5 protein-binding partners, including Hsp90 and calmodulin, increase Nox5 activity, whereas binding to Hsp70 and caveolin-1 decreases Nox5 activity (16). Proinflammatory transcription factors, nuclear factor κB (NF-κB), AP-1 (activator protein 1), and signal transducer and activator of transcription 1/3 (STAT1/STAT3), which increase [Ca^2+^]_i_, also regulate Nox5 expression in human aortic smooth muscle cells (74).

**Table 1.** Regulators of Nox5

*Stimulators*	*Inhibitors*
Nox5-binding proteins/chaperone proteins	
Calmodulin	Cav-1
Hsp90	Hsp70
	CHIP
Protein kinases	
PKCα	PKCɛ
CAM kinase II	
ERK1/2	
c-Abl	
c-Src	
Transcription factors	
NF-κB	
AP-1	
STAT1/STAT3	

AP-1, activator protein 1; CAM kinase II, Ca^2+^/calmodulin-dependent protein kinase II; ERK1/2, extracellular signal-regulated kinase1/2; NF-κB, nuclear factor κB; Nox, NADPH oxidase; PKC, protein kinase C; STAT, signal transducer and activator of transcription.

Nox5 is also modulated by other forms of post-translational modifications besides phosphorylation, including S-nitrosylation, SUMOylation (SUMO, small ubiquitin-related modifier), and palmitoylation (17). However, the significance of these different states of modification has yet to be elucidated.

## Tissue Distribution and Cell Expression of Nox5

The Nox5 gene is expressed in a variety of fetal tissues and in adult testes, spleen, lymph nodes, pancreas, placenta, bone marrow, uterus, kidney, stomach, and cancer cells. It is also present in cells of the cardiovascular system, including cardiomyocytes, and endothelial and vascular smooth muscle cells (18, 73). In the spleen, Nox5 is abundant in regions of mature B cells and T lymphocytes. The presence of Nox5 in circulating lymphocytes is controversial because original studies failed to identify Nox5 in circulating cells (76), whereas recent studies suggest that Nox5 regulates human monocyte differentiation into dendritic cells (75). Human monocytes and macrophages were also found to express Nox5; however, studies were performed mainly in human leukemia cell line cells (leukemia cell line) (75). The function of Nox5 is still unclear and what is known is indirect and based on Nox5 expression patterns. Nevertheless, growing evidence suggests an important role for Nox5 in cardiovascular and renal (patho)physiology, as highlighted below.

## Nox5 and Contraction

**FIG. 3.**
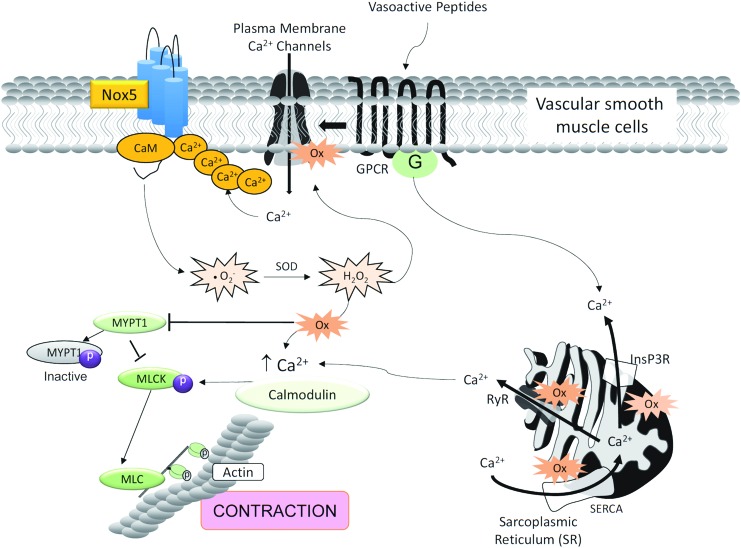
**Possible role of Nox5 in vascular contraction.** Nox5 may be a point of crosstalk between calcium and ROS, placing Nox5 as an important regulator of vascular contraction and development of vascular dysfunction in cardiovascular disease. Vasoactive factors, such as Ang II and ET-1, activate Nox5-induced ROS generation. Once ROS levels are increased in VSMCs, oxidation of calcium channels in the cell membrane or intracellular compartments, for example, ER, leads to dysregulated calcium influx and mobilization and activation of the contractile machinery—inhibition of MYPT1 and activation of MLCK and MLC, leading to contraction. Ang II, angiotensin II; ET-1, endothelin-1; MLC, myosin light chain; MLCK, myosin light chain kinase; MYPT1, myosin light chain phosphatase 1; VSMCs, vascular smooth muscle cells. To see this illustration in color, the reader is referred to the web version of this article at www.liebertpub.com/ars

Considering the importance of [Ca^2+^]_i_ in Nox5 regulation, and the critical role of Ca^2+^ in controlling the contractile machinery in muscle, we questioned whether Nox5 plays a role in vascular contraction. Our recent data show that in human vascular smooth muscle cells, Nox5 regulates procontractile signaling pathways, effects that are attenuated in siRNA Nox5-silenced human vascular smooth muscle cells (Fig. 3) (8, 81).

The possible role of Nox5 in contraction was previously highlighted in arthropod models, which express an ortholog of Nox5, called dNox (38, 99). Depletion of dNox in the musculature of Drosophila resulted in retention of mature eggs within ovaries, leading to female sterility (99). These effects were due to failure of ovarian muscles to contract in an agonist-dependent manner (99). Findings from this study highlighted the potentially important role of Nox5 in the regulation of smooth muscle contraction.

## Function of Nox5 in Vascular and Renal Systems

### Vascular Nox5

The expression and functional relevance of Nox5 and its isoforms in vessels remain unclear. In an attempt to address this, Pandey *et al.* (94) comprehensively characterized the expression and ROS-generating function of Nox5 and its splice variants in human arteries and veins. They found that mRNAs encoding Nox5α and Nox5β were present in isolated human internal mammary arteries and saphenous veins. However, unlike studies in cultured vascular cells, Nox5δ and Nox5γ were not detected in intact vessels and may reflect the absence of these Nox isoforms in blood vessels or possibly very low expression levels. Vascular Nox5α and Nox5β variants are catalytically active and generate ROS in both the endothelium and vascular media of arteries and veins.

In cultured human aortic endothelial cells, all Nox5 variants have been identified (107). However, only Nox5α and Nox5β seem to produce ROS (107). While Nox5δ, Nox5γ, and Nox5ɛ are expressed in cultured vascular cells, they are catalytically inactive, but associate with active Nox5 and function as dominant negatives by inhibiting ROS generation. In human microvascular endothelial cells, Nox5β and Nox5ɛ increased basal ROS levels, but in ionomycin-stimulated cells, only Nox5β was activated to generate O_2_^−^ (80). Differential expression of Nox5 variants in human endothelial cells may reflect cellular heterogeneity between the aorta and microvessels.

In cultured human endothelial cells, Nox5 is regulated by Ca^2+^ and calmodulin, but not by Rac1 (109). Nox5 inactivates NO signaling and promotes phosphorylation of ERK1/2, c-Jun N-terminal kinases, P38 mitogen-activated protein kinase, and Janus kinase 2, inducing apoptosis, proliferation, migration, and angiogenesis (80). Nox5 also plays a role in thrombin-induced actin cytoskeleton derangement, monocyte adhesion, and migration in endothelial cells, effects that are inhibited by Ang-(1–7) through downregulation of Nox5-induced ROS generation (93). In cultured human vascular smooth muscle cells, Nox5 stimulates MAP kinase signaling and Ca^2+^-activated K^+^ channels and induces cell proliferation and migration (37).

**FIG. 4.**
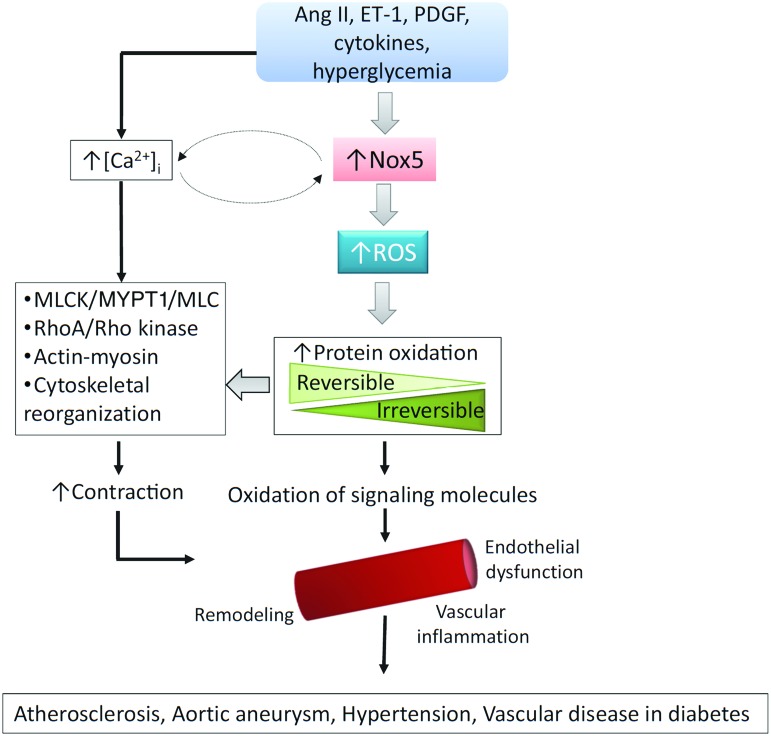
**Schematic demonstrating vascular signaling effects of Nox5.** Schematic demonstrating putative mechanisms whereby activation of Nox5 leads to vascular dysfunction, contraction, and injury in cardiovascular disease. Vasoactive peptides (Ang II and ET-1), growth factors, cytokines, and hyperglycemia induce Nox5 activation and increased levels of intracellular free Ca^2+^ ([Ca^2+^]_i_), which influence redox-sensitive and Ca^2+^-dependent signaling molecules associated with contraction, inflammation, growth, and endothelial function. Increased Nox5-mediated oxidative stress leads to increased protein oxidation (reversible and irreversible forms) and activation of signaling pathways that influence vascular function and structure in cardiovascular disease. PDGF, platelet-derived growth factor. To see this illustration in color, the reader is referred to the web version of this article at www.liebertpub.com/ars

Of the Nox isoforms present in human vessels, Nox5 seems to be the major ROS-generating oxidase (58). In human vascular cells, Nox5 is activated by Ang II, endothelin-1 (ET-1), tumor necrosis factor-α, and platelet-derived growth factor (PDGF) and it plays an important role in agonist-stimulated O_2_^−^ generation and redox signaling (80, 58) and has been implicated in vascular smooth muscle cell migration, proliferation, angiogenesis, inflammation, and contraction (Fig. 4). Human studies demonstrated increased vascular Nox5 expression in atherosclerosis, hypertension, myocardial infarction, and aortic aneurysm (58).

### Renal Nox5

Nox5 is expressed in adult human kidneys and is upregulated in chronic kidney disease, including diabetic nephropathy (53). Nox5 has been identified in renal endothelial cells, mesangial cells, podocytes, tubular epithelial cells, and interstitial fibroblasts (44). In human diabetic glomeruli, Nox5 expression was increased compared with nondiabetic glomeruli. In human podocyte cultures, Ang II increased Nox5-induced ROS production, effects that were attenuated in siRNA-mediated Nox5 knockdown (42). Nox5 silencing in podocytes was associated with altered cytoskeletal dynamics and a Rac-mediated motile phenotype, with impaired podocyte function (54). Nox5 is also expressed in human tubule cells. Nox5 expression and Nox activity were increased in renal proximal tubule cells from hypertensive patients compared with cells from normotensive counterparts (132). This differential Nox5 expression in hypertension was attributed to an abnormal renal dopaminergic system (57, 132). Nox5 may also be important in sepsis-induced acute kidney injury, where its expression is markedly increased (41). This seems to be regulated by miR-4321 (41).

## Insights from Mice Expressing the Human Nox5 Gene

**FIG. 5.**
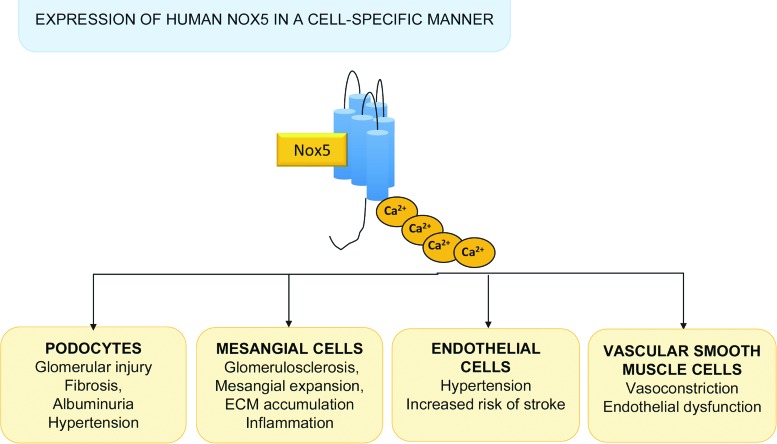
**Insights from animals expressing Nox5.** Studies in animals with inducible expression of human Nox5 in mice in a cell-specific manner demonstrated possible roles of Nox5 in the pathophysiology of cardiovascular diseases. Glomerular damage, kidney dysfunction, and hypertension were observed in mice expressing Nox5 specifically in podocytes. Nox5 expression in mesangial cells influenced processes related to fibrosis and inflammation. In endothelial cells, expression of Nox5 induced changes in blood pressure and increased risk of stroke. Nox5 in vascular smooth muscle cells is associated with vascular hypercontractility and endothelial dysfunction. To see this illustration in color, the reader is referred to the web version of this article at www.liebertpub.com/ars

Genes encoding isoforms of Nox5 have been identified in invertebrates and vertebrates (38, 98). However, for unknown reasons, the Nox5 gene was lost in rodents during evolution. Hence, experimental data from mice or rats cannot be extrapolated to human physiology or disease, posing major challenges in Nox5 research. To address this, we generated transgenic mice expressing human Nox5 in a cell-specific manner. Nox5 transgenic mice with podocyte-specific expression of human Nox5 exhibited increased renal ROS production, glomerular injury and tubulointerstitial fibrosis, albuminuria, and elevated blood pressure, phenomena that are exacerbated in the presence of diabetes (Fig. 5) (44, 53). These mice also had podocyte foot process effacement, suggesting a deleterious role of Nox5 in podocyte damage and development of albuminuria in diabetic nephropathy. In mice expressing human Nox5 in vascular smooth muscle cells/renal mesangial cells and made diabetic with streptozotocin, glomerular ROS production was increased and this was associated with accelerated glomerulosclerosis, mesangial expansion, and extracellular matrix protein (collagen IV and fibronectin) accumulation (57). In addition, these mice exhibited renal inflammation as evidenced by increased macrophage infiltration and expression of the proinflammatory chemokine monocyte chemoattractant protein 1. Together, these studies in humanized Nox5 mice suggest an important role for Nox5 in vascular injury and progression of nephropathy in diabetes.

Mice expressing human Nox5 in an endothelial-specific manner exhibited increased blood pressure and increased risk of stroke (Fig. 5) (61). This seemed to be especially evident in female mice. These studies suggested that targeting Nox5 may be vaso- and neuroprotective in ischemic stroke.

## Nox5, Coronary Artery Disease, and Atherosclerosis

One of the earliest studies suggesting a link between vascular NOX5 and human cardiovascular disease was performed in coronary arteries in patients undergoing heart transplantation (48). In patients with coronary artery disease, expression of Nox5, both at the mRNA and protein levels, was markedly increased compared with those patients without coronary artery disease. Moreover, the magnitude of Nox5 expression and ROS generation correlated with the severity of atherosclerosis (48). Nox5 has also been identified in the heart and is expressed in endothelial and vascular smooth muscle cells in intramyocardial vessels and in cardiomyocytes. In infarcted hearts, Nox5 expression is increased, especially in infarctions older than 12 h (50).

The close association between Nox5 and Ca^2+^ may be especially important in the heart and coronary circulation where cardiovascular contractility is highly regulated. Studies in porcine coronary arteries demonstrated that Nox5 plays an important role in regulating the potassium intermediate/small-conductance calcium-activated channel, subfamily N member 4 (KCNN4), which seems to be important for coronary artery smooth muscle cell phenotypic modulation, contraction, and progression of atherosclerosis (45). AP-1 seems to be the link between Nox5 and KCNN4 in coronary arteries.

## Nox5 and Hypertension

Although the exact role of Nox5 in the pathophysiology of human hypertension is unclear, findings show that mice expressing human Nox5 in the kidney and endothelium have elevated blood pressure, suggesting a potential role for Nox5 in blood pressure regulation (54, 57, 61, 132). The clinical relevance of this has recently been highlighted in a study searching for novel blood pressure-associated genes. In a genome-wide association study (GWAS) of 475,000 individuals, Nox5 was identified as an important blood pressure-associated gene, especially linked to systolic blood pressure (62). Comprehensive Nox5 phenotyping in patients with hypertension is needed to better clarify the potential importance of Nox5 as a pathophysiological factor in cardiovascular disease.

## Nox5 Beyond the Cardiovascular System

**FIG. 6.**
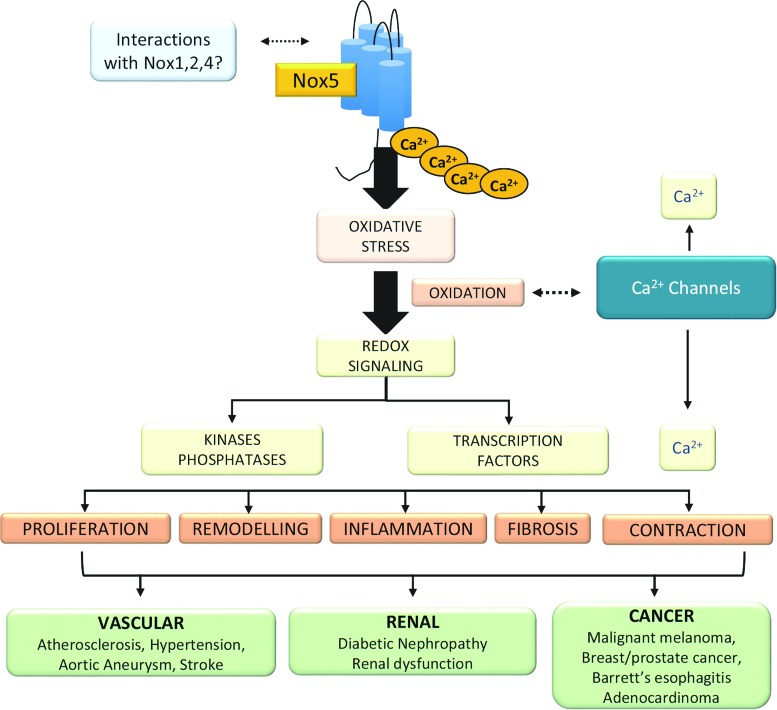
**Nox5 and human disease.** Nox5 is demonstrated to be a key regulator of calcium influx and signaling in many human cells. Nox5 regulates physiological signaling in cells from the cardiovascular, renal, and reproductive systems. Through ROS generation and oxidation, Nox5 influences signaling kinases, phosphatases, and transcription factors, resulting in contraction, inflammation, proliferation, fibrosis, and remodeling. These effects contribute to development of cardiovascular diseases, renal pathologies, and cancer. To see this illustration in color, the reader is referred to the web version of this article at www.liebertpub.com/ars

In addition to its potential role in cardiovascular (patho)physiology, Nox5 has been implicated to play an important role in various physiological and pathological processes (Fig. 6).

### Nox5 and regulation of sperm function

Nox5 seems to be critically involved in normal sperm function and motility, which are regulated by ROS (88). Nox5 is expressed in human spermatozoa and localizes in the flagella/neck region and acrosome. Stimulation of spermatozoa with a calcium ionophore, phorbol ester, or H_2_O_2_ increased sperm motility, effects that were dependent on Nox5-induced ROS generation (124, 125). These processes are regulated by the tyrosine kinase c-Abl and the Hv1 proton channel, which interact physically with Nox5 (2, 88).

### Nox5 and cancer

Growing evidence indicates that Nox-derived ROS generation, either constitutively or as a result of chronic inflammation or cell stress, plays a role in proliferation of cancer cells and tumor growth (1, 24, 102). Nox1, Nox4, and Nox5 have been identified in various types of cancers. Increased Nox activity promotes redox-mediated DNA damage, tissue injury, dysregulated cell proliferation, adhesion, angiogenesis, and tumor growth (102). Nox5 is highly expressed in malignant melanomas, prostate cancer, breast cancer, and Barrett's esophagus-associated adenocarcinomas (28, 34, 130). Signaling pathways implicated in Nox-ROS-associated cancer involve protein kinases of the MAPK cascade, phosphatidylinositol-3-kinase, and PKC and transcription factors, including apurinic/apyrimidinic endonuclease 1/redox effector factor 1, hypoxia-inducible factor 1α, AP-1, nuclear factor erythroid 2-related factor 2, tumor protein 53, forkhead box protein, STAT, and β-catenin (39, 55). Similar redox-sensitive pathways have been identified in vascular hypertrophy associated with hypertension and atherosclerosis (11, 65, 120).

## Conclusions

The importance of Noxs in ROS generation in the cardiovascular system and other systems is now clear. Nox isoforms are differentially regulated. Whereas Nox1, Nox2, and Nox4 have an obligatory need for p22phox for their activation to generate ROS, Nox5 is p22phox independent and produces O_2_^−^ in a constitutive and inducible manner. Experimental models have identified a role for Nox1, Nox2, and Nox4 in various cardiovascular pathologies, including hypertension, atherosclerosis, cardiovascular and renal complications of diabetes, and pulmonary hypertension. While there have been advancements in the molecular biology and biochemistry of Nox5, there is a paucity of information on its pathophysiological role in humans. This is reflected by the relatively few publications on Nox5 (164 original articles with “Nox5” as keyword since its discovery in 2001) and may relate, in part, to the lack of rodent experimental models that express Nox5. However, studies in transgenic mice that express human Nox5 and investigations in larger mammals that contain Nox5 endogenously, including rabbits and primates, will shed light on the physiological and pathological significance of Nox5. Moreover, studies in humans in health and disease will unravel the clinical role of Nox5. The recent GWAS highlighting the association between the Nox5 gene and hypertension is certainly interesting and provides a credible foundation for further investigation of Nox5 in human cardiovascular disease.

## Abbreviations Used

Ang II: angiotensin II
AP-1: activator protein 1
CAM kinase II: Ca^2+^/calmodulin-dependent protein kinase II
CGD: chronic granulomatous disease
ER: endoplasmic reticulum
ERK1/2: extracellular signal-regulated kinase1/2
ET-1: endothelin-1
GWAS: genome-wide association study
H_2_O_2_: hydrogen peroxide
KCNN4: potassium intermediate/small-conductance calcium-activated channel, subfamily N, member 4
NF-κB: nuclear factor κB
NO: nitric oxide
Nox: NADPH oxidase
PDGF: platelet-derived growth factor
PKC: protein kinase C
ROS: reactive oxygen species
STAT: signal transducer and activator of transcription
